# Evaluation of regenerated cartilage using T2 mapping methods after opening-wedge high tibial osteotomy with microfracture at the cartilage defect: a preliminary study

**DOI:** 10.1186/s40634-021-00413-3

**Published:** 2021-10-16

**Authors:** Ken Iida, Yusuke Hashimoto, Yohei Nishida, Shinya Yamasaki, Hiroaki Nakamura

**Affiliations:** 1 Department of Orthopaedic Surgery, Osaka City University Graduate School of Medicine, Osaka, Japan; 2 Department of Orthopaedic Surgery, Osaka City General Hospital, Osaka, Japan

**Keywords:** High tibial osteotomy, Opening wedge, Knee, Cartilage regeneration, T2 mapping

## Abstract

**Purpose:**

This study evaluated the regenerated cartilage after opening-wedge high tibial osteotomy (OWHTO) with concomitant microfracture by second-look arthroscopy, Magnetic Resonance Observation of Cartilage Repair Tissue (MOCART) score and magnetic resonance imaging (MRI) T2 mapping. It was hypothesised that cartilage regeneration can be achieved by HTO, but the quality of regenerated cartilage is not normal cartilage.

**Methods:**

OWHTO was performed in eight knees of seven patients (mean age, 57.6 ± 5.2 years). Microfracture for the cartilage defect was performed followed by OWHTO, and second-look arthroscopy was performed at the time of plate removal (14.1 ± 4.5 months after OWHTO). MRI was assessed at three months and one year after surgery. The status of articular cartilage regeneration was assessed by the ICRS grade, MOCART score and T2 value.

**Results:**

The number of subjects in ICRS grade 1/2/3/4 changed significantly from 0/0/4/4 preoperatively to 0/2/6/0 postoperatively in the medial femoral condyle (MFC) (*P* < 0.05) and 0/0/0/8 preoperatively to 0/0/7/1 postoperatively in the medial tibial plateau (MTP) (*P* < 0.05). Mean MOCART scores for MFC and MTP at one year after surgery exhibited significant increases compared with the results at three months after surgery. Mean T2 values for MFC and MTP did not differ at three months and one year after surgery.

**Conclusion:**

The appearance and morphological evaluation by ICRS grade and MOCART score of regenerated cartilage were improved after OWHTO with concomitant microfracture. However, there were no significant qualitative differences in T2 values. This suggests that the regenerated cartilage tissue was likely to be insufficient cartilage.

**Level of evidence:**

Level IV, therapeutic case series.

## Introduction

High tibial osteotomy (HTO) is appropriate for the treatment of medial knee osteoarthritis (OA) with varus deformity in patients who need sufficient pain relief [9, 18, 19]. Regeneration of articular cartilage after HTO was described in 1979 by several authors [7]. Although cartilage repair can be achieved by HTO without microfracture in medial compartment OA, microfracture is commonly performed in conjunction with HTO by orthopaedic surgeons because of its favourable outcomes, minimal risk and rapidity [11, 21]. The obtained defects are filled by mesenchymal stem cells and progenitor cells, resulting in the formation of new cartilage [6]. Repair tissue after microfracture has mainly been reported to be fibrocartilage, whereas the repair tissue after cartilage transplantation procedures has more often been reported to be hyaline-like [1]. The cartilage repair tissue after microfracture and abrasion was found to be fibrocartilage on biopsy, but biopsy can be a highly invasive test. Magnetic resonance imaging (MRI) is a minimally invasive method used for the assessment of cartilage. To measure the morphologic integration and biochemical constitution of cartilage repair tissue noninvasively, MRI is the method of choice, and a validated scoring system for the evaluation of cartilage repair sites is the Magnetic Resonance Observation of Cartilage Repair Tissue (MOCART) system [5]. Quantitative MRI examination techniques have recently been developed for the assessment of cartilage lesions. Quantitative T2 mapping provides information about the interactions of water molecules and the collagen network within the articular cartilage [16, 4]. This study was performed to evaluate MRI T2 mapping for assessment of cartilage regeneration after opening-wedge HTO (OWHTO) with concomitant microfracture, with the healing status being confirmed by arthroscopy and MOCART score. Our hypothesis was that cartilage regeneration would be induced by OWHTO according to the International Cartilage Repair Society (ICRS) grade and MOCART score, but there would be no significant differences in T2 relaxation times because the regenerated cartilage would contain fibrocartilage tissue.

## Materials and methods

The study was approved by the hospital ethics committee and internal review board at our institution. Informed consent was obtained from all participants included in the study. Thirteen patients who underwent OWHTO with microfracture at the Department of Orthopaedic Surgery at Osaka City University Hospital from 2013 to 2019 were enrolled retrospectively. The inclusion criteria for the study were: patients who satisfied our surgical indications, comprising painful OA localized in the medial compartment of the knee with no functional instability of the anterior cruciate ligament. The exclusion criteria were: patients with insufficient information for review and patients who did not undergo implant removal and second-look arthroscopy. Finally, a total of seven patients (eight knees) were enrolled in the study (median age, 57 years; range, 51–66 years). Microfracture was performed followed by OWHTO, and a second-look arthroscopy was performed at the time of plate removal (14.1 ± 4.5 months after OWHTO).

### Surgical procedure

The patients were placed in the supine position. An initial arthroscopic examination was routinely performed prior to OWHTO. After evaluating the cartilage status in each compartment, accurate debridement was performed for all unstable and damaged cartilage in the lesion, including the calcified layer down to the subchondral bone. All loose or marginally attached cartilage was debrided from the surrounding rim of the defect to form (Fig. 3a). An arthroscopic awl was then used to make multiple holes in the defect at 3–4 mm apart (Fig. 3b) on the medial femoral condyle (MFC) and medial tibial plateau (MTP). OWHTO was performed using an opening-wedge technique with rigid plate fixation. The rigid plating was performed using TOMOFIX (Synthes US) and Tris (ORINPUS Japan). The amount of angular correction was planned preoperatively, aiming to achieve a 172° femorotibial angle on a one-leg standing radiograph postoperatively. The femorotibial angle was defined as the lateral angle between the anatomical axes of the femur and the tibia measured on a standing anteroposterior radiograph as reported previously [2]. The target mechanical axis was set at 62.5% from the medial edge of the tibial plateau to its entire width as described by Fujisawa et al. [7]. The articular cartilage was re-examined postoperatively by arthroscopy at the time of plate removal (Fig. 2c). The grade of cartilage injury associated with degeneration was recorded preoperatively and postoperatively in each compartment according to the ICRS classification [3].

### Postoperative rehabilitation

Patients started a postoperative rehabilitation program, including isometric quadriceps exercises and range of motion exercises, and partial weight-bearing exercise on the day after surgery. All patients had a regular continuous passive motion machine applied for two weeks from the second postoperative day and quadriceps strengthening exercises were taught by the medical team. Full weight-bearing exercise was permitted at four weeks postoperatively.

### Clinical and imaging examinations

**Fig. 1:**
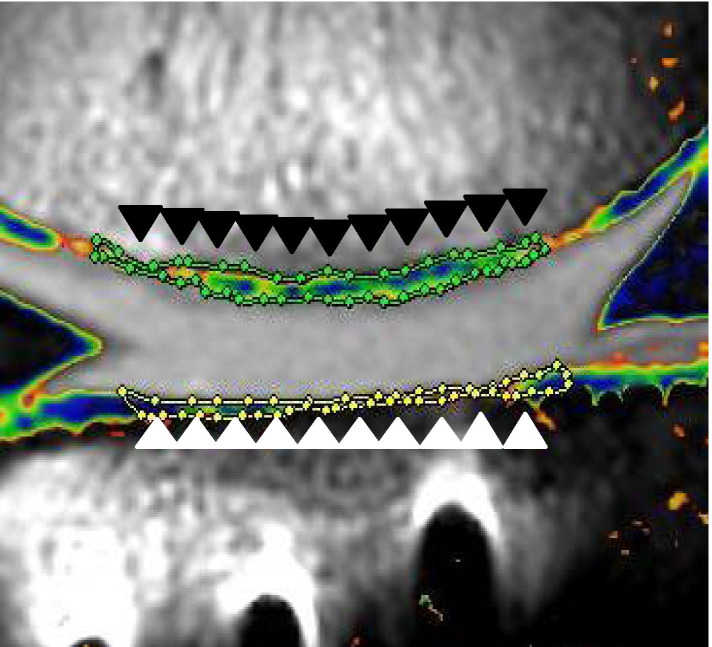
Regions of interest (ROIs) were drawn around the repair site. The ROIs for measurement of T2 relaxation times in the lateral femoral condyle (black arrowhead) and tibial plateau (white arrowhead) are shown

For the clinical evaluation, the IKDC score and Lysholm score were used to determine joint function before surgery and at one year after surgery [10, 13]. Lower limb alignment was evaluated by the femorotibial angle and mechanical axis, defined as the lateral angle between the anatomical axes of the femur and tibia, measured on standing anteroposterior radiographs before surgery and at one year after surgery. All patients were scanned in the supine position with a 3.0-T MRI system (Achieva; Philips) at three months and one year after surgery. The knee joint of each patient was centred in an 8-channel SENSE knee coil (Philips). The morphology and signal change in each meniscus were examined with a proton density-weighted multislice turbo spin echo sequence with fat saturation using the following settings: repetition time, 3462 ms; echo time (TE), 10 ms; receiver band width, 217.4 Hz; echo train length, 18; field of view, 16 × 16 cm; matrix, 416 × 416; slice thickness, 3 mm; slice gap, 0.3 mm; number of slices, 26; number of excitations, 2. For the T2 mapping, sagittal T2-weighted images with six echoes were obtained with a multislice turbo spin echo sequence using the following settings: repetition time, 2100 ms; TE, 10, 20, 30, 40, 50 and 60 ms; receiver band width, 290.7 kHz; echo train length, 18; field of view, 16 × 16 cm; matrix, 352 × 352; slice thickness, 3 mm; slice gap, 0.3 mm; number of slices, 26; number of excitations, 1; total scan time, 14 min. Quantitative and qualitative assessments of the repair tissue were carried out at three months and one year after surgery on follow-up MRI using the MOCART scoring system and T2 mapping. The MOCART score was evaluated to perform a structured morphological assessment of the articular cartilage repair. A MOCART score of 100 was the best possible MRI outcome and a score of 0 was the worst possible outcome [15]. For the T2 mapping, the T2 relaxation time of each pixel was calculated using the monoexponential curve-fitting method of AZE Virtual Place (AZE Ltd.). The region of interest (ROI) for the measurement of T2 relaxation times was set according to the area of articular cartilage showing high signal intensity on the proton density image. T2 relaxation times were calculated based on the exponential decay of the T2 relaxation. In one slice, several ROIs were defined, and one ROI encircled the repair site. The high signal intensity indicated cartilage regeneration, and one sagittal plane slice showing the high signal was included for the lateral femorotibial compartment (Fig. 1). Two surgeons (K.I. and Y.N.) assessed all standard cases twice, with a four-week interval, to test the interobserver and intraobserver reliabilities of the MRI assessments. Reliability of the measurements was assessed by examining the interobserver and intraobserver reliabilities using the intraclass correlation coefficient (ICC). A measurement was considered reliable if the ICC was > 0.80. The interobserver and intraobserver reliabilities were satisfactory, and the mean values were 0.815 and 0.897, respectively.

### Statistical methods

The primary dependent variables were the IKDC score and Lysholm score at one year after surgery for clinical outcomes, and the femorotibial angle and mechanical axis as radiographic outcomes. The Wilcoxon signed-rank test was performed for evaluation of changes between the preoperative and one-year postoperative values. Comparisons of the preoperative and postoperative ICRS grades of the articular cartilage were carried out using the Pearson chi-square test. Comparisons of the continuous variables of MOCART scores and T2 values between three months and one year after surgery were investigated using the Mann–Whitney U test. All analyses were conducted with significance defined as *P* < 0.05. A power analysis was performed with the power (α) difference and standard deviations set at 0.8, 0.05, 26.4 and 10.1, respectively, according to the T2 values of the MOCART scores of the MFC. The analysis revealed that a minimum of four knees were required for the Wilcoxon test to detect a difference between the results at three months after surgery and one year after surgery. EZR software (version 1.38; Saitama Medical Center, Jichi Medical University, Saitama, Japan) was used throughout.

## Results

### Clinical and radiological outcomes

**Fig. 2:**
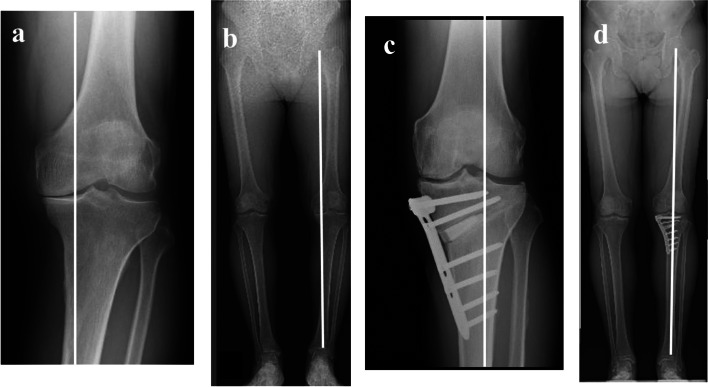
**a**, **b** Preoperative radiographs showing varus correction: the mechanical axis passes through 35.1% and the FTA is 181° in the left leg. **c**, **d** Postoperative radiographs showing that the mechanical axis passes through 62.1% and the FTA is 171° in the left leg

Table 1 summarizes the patient characteristics and clinical, arthroscopic and radiographic results in all study patients. The mean IKDC and Lysholm scores were significantly improved at one year after surgery compared with the preoperative values (*P* < 0.05). The femorotibial angle was 180.7° ± 1.4° (mean ± SD) preoperatively and 172.1° ± 1.2° postoperatively. The mean mechanical axis changed from 35.6% ± 1.3% preoperatively to 63.5% ± 1.0% postoperatively. Figure 2 shows representative X-rays taken preoperatively and postoperatively.

**Table 1:** Patient characteristics and arthroscopic and radiographic results in all patients

Variable	Value
Gender (female/male)	0/8
Age (years)	57.6 ± 5.2 (53–66)
Height (cm)	164,6 ± 7.3 (155–178)
Weight (kg)	72.7 ± 5.6 (67–81)
Body mass indexa	26.8 ± 1.8(24.6–30.4)
Preoperative FTA (°)	180.7 ± 1.4
Post-operative FTA (°)	172.1 ± 1.2
Preoperative %MA (%)	35.6 ± 1.3
Post-operative %MA (%)	63.5 ± 1.0
Preoperative Lysholm score	63.5 ± 6.5
Postoperative Lysholm score	87.2 ± 9.2
Preoperative IKDC score	47.2 ± 8.3
Postoperative IKDC score	66.5 ± 13.7
Articular cartilage degeneration grade in the MFC at HTO (1/2/3/4)	0/0/4/4
Articular cartilage degeneration grade in the MFC at implant removal (1/2/3/4)	0/2/6/0
Articular cartilage degeneration grade in the MTP at HTO (1/2/3/4)	0/0/0/8
Articular cartilage degeneration grade at in the MTP implant removal (1/2/3/4)	0/0/7/1

Articular cartilage degeneration grade was classified according to the arthroscopic grading system of the

International Cartilage Repair Society

FTA femorotibial angle, %MA percentage of mechanical axis, HTO high tibial osteotomy, MFC medial femoral condyle, MTP medial tibial plateau

Values are expressed as mean ± standard deviation, with range in parentheses

### Arthroscopic assessment of articular cartilage

**Fig. 3:**
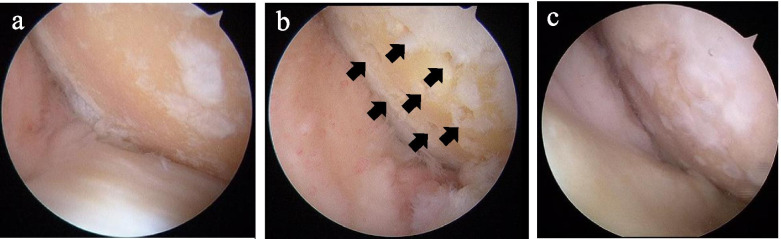
Arthroscopic views. **a** Full-thickness articular cartilage defect of the lateral femoral condyle. **b** Microfracture (black arrows) for a cartilage defect in the lateral femoral condyle. c) Regenerated cartilage of the lateral femoral condyle

As shown in Table 2, cases with cartilage loss were classified as ICRS grade 4 at the time of initial arthroscopy, while cases with partial coverage with white fibrocartilage were classified as ICRS grade 3 at the time of second-look arthroscopy (Fig. 3).

**Table 2:** Frequency of ICRS grade in articular cartilage

Grade	Preoperative	Postoperative	*P value
MFC			0.03
2	0	2	
3	4	6	
4	4	0	
MTP			
3	0	7	< 0.01
4	8	1	

Values are given as number of knees

MFC medial femoral condyle, MTP medial tibial plateau

*P values were determined by Pearson’s chi-square test

### MRI outcomes

**Fig. 4:**
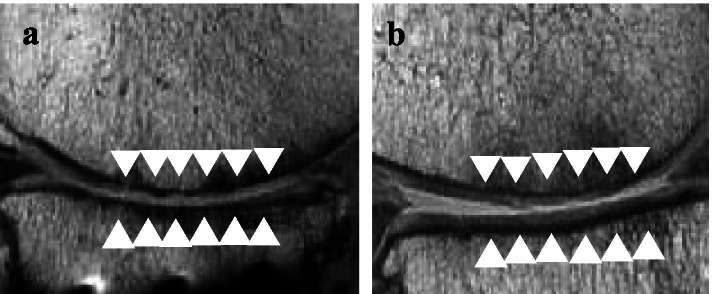
**a** Sagittal images of the knee at three months postoperatively. The filling of the defect was incomplete, the repair tissue surface was damaged, the subchondral bone was not intact (arrows) and the MOCART score was 40 points. **b** Follow-up sagittal images at one year postoperatively. Incomplete filling of the defect along with incomplete integration with the adjacent native cartilage (arrows) was observed and the MOCART score was 60 points

The MOCART scores and T2 values in the repaired cartilage are summarized in Table 3. The mean MOCART scores of the MFC at three months and one year after surgery were 42.8 ± 4.8 and 69.2 ± 10.1, respectively, and the mean MOCART scores of the MTP were 49.2 ± 1.8 and 70.0 ± 9.5, respectively. The mean MOCART scores of the MFC and MTP at one year after surgery showed significant increases compared with the results at three months after surgery (*P* < 0.05). The mean T2 values of the MFC at three months and one year after surgery were 52.7 ± 4.6 ms and 51.2 ± 5.1 ms, respectively, and the mean T2 values of the MTP were 49.4 ± 4.0 ms and 48.3 ± 3.4 ms, respectively. There were no differences at three months and one year after surgery. Figure 4 shows representative MRI images taken at three months and one year after surgery.

**Table 3:** MOCART scores and T2 values of the repaired cartilage

Variable’s	Score	three mo Postoperatively		one yr Postoperatively	
		MFC n(%)	MTP n(%)	MFC n(%)	MTPn(%)
**1. Degree of defect repair and filling of the defect**					
**Complete**	**20**			**2(25)**	**1(12.5)**
**Hypertrophy**	**15**			**3(37.5)**	**3(37.5)**
**Incomplete**					
**< 50% of adjacent cartilage**	**10**	**2(25)**	**7(87.5)**	**3(37.5)**	**4(50)**
**> 50% of adjacent cartilage**	**5**	**6(75)**	**1(12.5)**		
**Subchondral bone exposed**	**0**				
**2. Integration to border zone**					
**Complete**	**15**			**1(12.5)**	**1(12.5)**
**Incomplete**	**10**	**3(37.5)**	**2(25)**	**6(75)**	**6(75)**
**Defect visible**					
**< 50% of the length of the repair tissue**	**5**	**5(62.5)**	**5(62.5)**	**1(12.5)**	
**> 50% of the length of the repair tissue**	**0**		**1(12.5)**		
**3. Surface of the repair tissue**					
**Surface intact**	**10**				
**Surface damaged**					
**< 50% of repair tissue depth or total degeneration**	**5**	**8(100.0)**	**7(87.5)**	**8(100.0)**	**8(100.0)**
**> 50% of repair tissue depth or total degeneration**	**0**		**1(12.5)**		
**4. Structure of the repair tissue**					
**Homogenous**	**5**	**1(12.5)**	**1(12.5)**	**2(25)**	**2(25)**
**Inhomogenous or cleft formation**	**0**	**7(87.5)**	**7(87.5)**	**6(75)**	**6(75)**
**5. Signal intensity of the repair tissue**					
**Normal**	**30**				
**Nearly normal**	**15**	**1(12.5)**	**1(12.5)**	**1(12.5)**	**2(25)**
**Abnormal**	**0**	**7(87.5)**	**7(87.5)**	**7(87.5)**	**6(75)**
**6. Subchondral lamina**					
**Intact**	**5**	**3(37.5)**	**3(37.5)**	**6(75)**	**7(87.5)**
**Not intact**	**0**	**5(62.5)**	**5(62.5)**	**2(25)**	**1(12.5)**
**7. Subchondral bone**					
**Intact**	**5**	**5(62.5)**	**6(75)**	**6(75)**	**5(62.5)**
**Edema**	**0**	**3(37.5)**	**2(25)**	**2(25)**	**3(37.5)**
**8. Adhesions**					
**No**	**5**	**7(87.5)**	**7(87.5)**	**8(100.0)**	**7(87.5)**
**Yes**	**0**	**1(12.5)**	**1(12.5)**		**1(12.5)**
**9. Effusion**					
**No**	**5**	**6(75)**	**5(62.5)**	**7(87.5)**	**7(87.5)**
**Yes**	**0**	**2(25)**	**3(37.5)**	**1(12.5)**	**1(12.5)**
**Total**		**42.8 ± 4.8**	**49.2 ± 1.8**	**69.2 ± 10.1**^a^	**70.0 ± 9.5**^a^
**T2 Value**		**52.7 ± 4.6**	**49.4 ± 4.0**	**51.2 ± 5.1**	**48.3 ± 3.4**
^a^**statistical significance was set at p < 0.05.**					

Values are presented as number except for the total score as indicated

^a^Derived using the Mann–Whitney U test

## Discussion

One of the strengths of our study was that cartilage regeneration after OWHTO was significantly improved at one year after surgery according to the ICRS grade and MOCART score, but there were no significant differences in T2 relaxation times in terms of qualitative improvements. These results indicate that the regenerated cartilage tissue was likely to be insufficient cartilage.

Microfracture was performed according to the technique published by Steadman et al. as an arthroscopic procedure [20]. Using this method, the subchondral bone was penetrated to allow fibrin clot formation within the defect and the subsequent maturation of repair tissue, which fills the cartilage defect. The present study demonstrated that the rate of regenerative change with newly formed cartilaginous tissue was significantly higher on the surface with full-thickness cartilage loss and exposed subchondral bone. When ideal correction was obtained, the ulcerated lesion was thoroughly covered with fibrous and membranous tissue at 1.5–2 years after osteotomy [17, 7]. It has been reported that regeneration of the articular cartilage defined as partial or total coverage with fibrocartilage after closing-wedge HTO was achieved in 133 of 146 cases (91%) by Koshino et al. [14]. In this study, there were seven cases (87.5%) with cartilage repair. The frequency of cartilage repair using the ICRS grade was almost the same as that in the previous study.

In terms of the MOCART score after HTO, Verdonk et al. [22] reported that complete integration to the border zone was found in 25% of patients at two years after surgery. However, Kim et al. [12] reported complete integration in 1 of 14 patients with microfracture alone at the one-year follow-up. Our study also showed that complete integration with the border zone was found in 12.5% of patients at one year after surgery. Kim et al. [12] reported no hypertrophy of the cartilage and found subchondral bone marrow oedema in 78.6% of patients with OWHTO plus microfracture. Conversely, in our study, hypertrophy of the cartilage was observed in 37.5% of MFC and MTP, while subchondral bone marrow oedema was found in only 25% of MFC and 37.5% of MTP after OWHTO with concomitant microfracture. These different results may arise because the follow-up period in our study was one year, which was markedly shorter than the two-year follow-up in the study by Kim et al. [12].

In a recent study by White et al. [23], normal hyaline cartilage and cartilage repair tissue were found to be different in horses. Arthroscopic microfracture was performed, and evaluation of T2 values showed a significant trend across cartilage depth for low T2 values near the subchondral bone and higher T2 values near the cartilage surface. Arthroscopic evaluation confirmed the findings obtained by T2 mapping, namely complete filling of the microfracture and repair tissue sites. Previous histological studies on biopsy specimens from the cartilage site at which microfracture was performed indicated that the defect was repaired with fibrocartilage [8, 11]. However, evaluation of the repair tissue after microfracture using T2 mapping revealed that the T2 values of the repair tissue in knees with microfracture had a significantly lower value after more than one year compared with the normal cartilage [20]. There were no significant differences in the T2 values of cartilage repair tissue between three months and one year after surgery in this study. The mean T2 values were equal to those in the previous studies. This means that the regenerated cartilage after microfracture was fibrocartilage, and that it cannot be determined to be completely healed by qualitative assessment and instead healed as a mature scar.

The present study has some limitations. First, the number of cases was small and the follow-up period was relatively short. For more accurate evaluation of degenerated articular cartilage, a study with a larger number of cases and a longer follow-up period is required. Although a second-look arthroscopy was performed at the time of plate removal, the timing of the plate removal after HTO should be about one year after surgery, and therefore we inevitably performed cartilage evaluation at one year after surgery. Second, the study did not have reference values for normal cartilage. Third, we cannot exclude a possible effect of the metallic implants on the T2 relaxation times. However, the evaluation of the repair tissue using T2 mapping was performed over time following OWHTO, and thus the T2 relaxation times may not have been affected by the metallic implants.

## Conclusion

This study demonstrated that cartilage repair is induced following OWHTO according to the ICRS grade and MOCART score, but showed that there were no significant differences in T2 relaxation times to indicate qualitative improvements.
